# Expression and clinical significance of LAG-3, FGL1, PD-L1 and CD8^+^T cells in hepatocellular carcinoma using multiplex quantitative analysis

**DOI:** 10.1186/s12967-020-02469-8

**Published:** 2020-08-06

**Authors:** Mengzhou Guo, Feifei Yuan, Feng Qi, Jialei Sun, Qianwen Rao, Zhiying Zhao, Peixin Huang, Tingting Fang, Biwei Yang, Jinglin Xia

**Affiliations:** 1 Liver Cancer Institute, Zhongshan Hospital, Fudan University, 180 Fenglin Road, Shanghai, 200032 China; 2 Minhang Hospital, Shanghai Medical School of Fudan University, Shanghai, 201100 China

**Keywords:** LAG-3, FGL1, PD-L1, Hepatocellular carcinoma, Prognosis, Multiplex immunofluorescence staining

## Abstract

**Background:**

Fibrinogen-like protein 1 (FGL1)—Lymphocyte activating gene 3 (LAG-3) pathway is a promising immunotherapeutic target and has synergistic effect with programmed death 1 (PD-1)/programmed death ligand 1 (PD-L1). However, the prognostic significance of FGL1-LAG-3 pathway and the correlation with PD-L1 in hepatocellular carcinoma (HCC) remain unknown.

**Methods:**

The levels of LAG-3, FGL1, PD-L1 and cytotoxic T (CD8^+^T) cells in 143 HCC patients were assessed by multiplex immunofluorescence. Associations between the marker’s expression and clinical significances were studied.

**Results:**

We found FGL1 and LAG-3 densities were elevated while PD-L1 and CD8 were decreased in HCC tissues compared to adjacent normal liver tissues. High levels of FGL1 were strongly associated with high densities of LAG-3^+^cells but not PD-L1. CD8^+^ T cells densities had positive correlation with PD-L1 levels and negative association with FGL1 expression. Elevated densities of LAG-3^+^cells and low levels of CD8^+^ T cells were correlated with poor disease outcome. Moreover, LAG-3^+^cells deteriorated patient stratification based on the abundance of CD8^+^ T cells. Patients with positive PD-L1 expression on tumor cells (PD-L1 TC^+^) tended to have an improved survival than that with negative PD-L1 expression on tumor cells (PD-L1 TC^−^). Furthermore, PD-L1 TC^−^ in combination with high densities of LAG-3^+^cells showed the worst prognosis, and

PD-L1 TC^+^ patients with low densities of LAG-3^+^cells had the best prognosis.

**Conclusions:**

LAG-3, FGL1, PD-L1 and CD8 have distinct tissue distribution and relationships with each other. High levels of LAG-3^+^cells and CD8^+^ T cells represent unfavorable and favorable prognostic biomarkers for HCC respectively.

## Background

Hepatocellular carcinoma (HCC) is an extremely malignant tumor with the fourth leading cause of cancer mortality and ranks sixth in incidence worldwide [1]. Roughly 70% of HCC patients are diagnosed at intermediate or advanced stage with limited treatment options [2]. Frontline therapies like sorafenib or lenvatinib can only modestly prolong overall survival (OS) by about 1 year in advanced HCC, and with limited duration of benefits because of relatively high drug resistance [3, 4]. Immunotherapies have represented a main breakthrough in the treatment paradigm for oncology. Agents targeting the programmed cell death protein-1 (PD-1)/programmed death ligand-1 (PD-L1) immune checkpoint have showed promising efficacy and good safety profiles in various types of malignancy [5, 6]. Nivolumab and pembolizumab have received Food and Drug Administration (FDA) approval as second-line treatments for advanced HCC based on the Checkmate 040 [7] and Keynote 224 Trials [8]. However, subsequent phase III trials have failed to demonstrate statistically significant survival improvement in either first-line (nivolumab vs. sorafenib) or second-line (pembrolizumab vs. placebo) setting [9, 10]. Therefore, identification of other pivotal immune checkpoints and clarification the relationships of these immunotherapeutic targets in the tumor microenvironment (TME) are needed.

Lymphocyte activating gene 3 (LAG-3 or CD233) may be another promising immune checkpoint belonging to the immunoglobulin superfamily, which is expressed on tumor infiltrating lymphocytes (TILs) [11], natural killer cells [12], B cells [13] and dendritic cells [14]. LAG-3 has high structural homology with CD4 but greater capacity to bind major histocompatibility complex-II (MHC-II) molecules [12]. Alternative ligands, such as Galectin-3, LSECtin, and a-synuclein fibers, have been discovered to explain the inhibitory effects of LAG-3 on various types of lymphocytes without the engagement of MHC-II [15–17]. Recent study has demonstrated that fibrinogen-like protein 1 (FGL1) is a new major ligand for LAG-3, and blocking the FGL1-LAG-3 pathway can stimulate tumor immunity and inhibit tumor growth [18]. LAG-3 is significantly associated with prognosis and clinicopathological characteristics in various types of cancer [19], and has synergistic effects with PD-1/PD-L1 [20–23].

Despite being studied in many ongoing clinical trials, the expression of FGL1-LAG-3 pathway and the relationship with PD-L1 have not been clearly defined in HCC. Here, we used multiplex immunofluorescence to assess the distribution and clinical significance of LAG-3, FGL1, PD-L1 and cytotoxic T (CD8^+^T) protein expression in HCC.

## Methods

### Patients and tissue microarrays

161 pairs of HCC samples and matched non-tumor liver tissues were collected from patients who underwent hepatectomy at Zhongshan Hospital of Fudan University between November 2005 and December 2012, but 17 pairs of cases were excluded due to lack of follow-up data and 1 pair of case was excluded because of deficiency of tumor compartment (< 5%). Two cores were taken from representative tissue areas of each case (tumor tissue and paired liver tissue adjacent to the tumor within a distance of 10 mm) to construct tumor tissue microarrays (TMAs), which was described previously [24]. Then we applied H&E staining on the 2 TMAs to validate pathology type of each tissue on each TMA, and the results of H&E staining was shown in Additional file 1: Figure S1. The histopathological and clinical staging classification were carried out according to the 7th AJCC Tumor Node Metastasis (TNM) Staging and Barcelona Clinic Liver Cancer (BCLC) staging system respectively. Clinicopathologic characteristics of all patients are summarized in Table 1. Informed consents were obtained from each patient and the study was approved by the Zhongshan Hospital Ethics Committee.

**Table 1:** Relationship between immune cell markers and clinicopathological features (n = 143)

Variable	LAG-3			CD8			PD-L1 TC			PD-L1 IC			FGL1 TC			FGL1 IC		
	Low	High	*P*	Low	High	*P*	Negative	Positive	*P*	Negative	Positive	*P*	Negative	Positive	*P*	Negative	Positive	*P*
Patients	83	60		53	90		91	52		60	83		84	59		113	30	
Age(years)			0.137			0.512			0.366			0.166			0.313			0.008
> 50	32	16		16	32		58	37		36	59		53	42		69	26	
≤ 50	51	44		37	58		33	15		24	24		31	17		44	4	
Gender			0.192			0.163			0.182			0.428			0.827			0.890
Male	13	5		4	14		77	48		54	71		73	52		99	26	
Female	70	55		49	76		14	4		6	12		11	7		14	4	
HbsAg			0.627			0.595			0.642			0.608			0.936			0.540
Negative	12	7		6	13		13	6		9	10		11	8		14	5	
Positive	71	53		47	77		78	46		51	73		73	51		99	25	
Cirrhosis			0.077			0.565			0.475			0.172			0.002			0.284
No	46	42		31	57		54	34		33	55		43	45		67	21	
Yes	37	18		22	33		37	18		27	28		41	14		46	9	
AFP(ng/ml)			0.189			0.656			0.340			0.696			0.245			0.813
≤ 20	41	23		25	39		38	26		28	36		41	23		50	14	
> 20	42	37		28	51		53	26		32	47		43	36		63	16	
Vascular invasion			0.231			0.783			1.000			0.179			1.000			1.000
Absent	79	53		48	84		84	48		58	74		78	54		102	28	
Present	4	7		5	6		7	4		2	9		6	5		11	2	
Tumor size (cm)			0.652			0.145			0.008			0.501			0.141			0.102
≤ 5	58	44		34	68		58	44		41	61		56	46		77	25	
> 5	25	16		19	22		33	8		19	22		28	13		36	5	
Tumor number			0.381			0.329			0.899			0.975			0.618			0.103
Single	71	48		42	77		76	43		50	69		71	48		97	22	
Multiple	12	12		11	13		15	9		10	14		13	11		16	8	
Tumor differentiation			0.489			0.289			0.515			0.452			0.196			0.675
I-II	75	52		49	78		82	45		54	73		77	50		101	26	
III	8	8		4	12		9	7		6	10		7	9		12	4	
BCLC stage			0.668			0.071			0.054			0.701			0.643			0.321
0 + A	50	34		26	58		48	36		36	48		48	36		64	20	
B + C	33	26		27	32		43	16		24	35		36	23		49	10	

*P* < 0.05 was considered statistically significant, Pearson *χ*^2^ tests

*HBsAg* hepatitis B surface antigen, *AFP* αfetoprotein, *BCLC* Barcelona Clinic Liver Cancer, *TC* tumor cells, *IC* immune cells

### Multiplex immunofluorescence staining

Multiplex immunofluorescence (mIF) with antibodies specific for FGL1, LAG-3, PD-L1, CD8 and cytokeratin 18 were performed on these tissues according to protocols have been described and validated [25]. Briefly, the slides were underwent deparaffinization in xylene, and then rehydrated by ethanol. Antigen retrieval (AR) was performed in Tris–EDTA buffer (PH9.0) at boiling point for 15 min, and endogenous peroxidase activity was blocked with 3% hydrogen peroxide for 15 min at room temperature. Non-specific antigens were blocked with goat serum solution for 30 min. The slides were incubated with primary antibodies overnight at 4 ℃ (Detailed information of the primary antibody was presented in Additional file 2: Table S1), followed by the addition of horseradish peroxidase (HRP)-conjugated secondary antibody (Ab) at room temperature for 30 min. Next, the slides were incubated with Opal tyramide signal amplification (TSA) Fuorochromes (Opal 7-Color Manual IHC Kit, Perkin Elmer, NEL811001KT) for 20 min at 37 ºC. Between subsequent each staining runs, slides were microwaved to strip the Ab-TSA complex and blocked with goat serum solution. On the last run, 4′,6-diamidino-2-phenylindole, dihydrochloride (DAPI) was applied for visualizing nuclei, and mounted with glycerine.

### Imaging, quantification and scoring

Slides were scanned using the Vectra 3.0 imaging system Automated Quantitative Pathology Imaging System (PerkinElmer). After scanning, Images were then unmixed and analyzed to quantify the expression of each immune marker on individual cells (defined by nuclei staining [DAPI]) in the tumor and stromal compartments using inform software. Marker colocalization was used to identify populations of CD8^+^, tumor cells (TC, Cytokeratin18^+^), immune cells (IC, non-tumor cells, Cytokeratin18^−^), TC expressing PD-L1 (Cytokeratin18^+^PD-L1^+^), TC expressing FGL1 (Cytokeratin18^+^FGL1^+^), IC expressing PD-L1 (Cytokeratin18^−^PD-L1^+^), IC expressing FGL1(Cytokeratin18^−^FGL1^+^). FGL1 and PD-L1 expression were evaluated separately for TC and IC. For TC, the proportion of FGL1 or PD-L1-positive TC among total TC were quantified. For IC, the percentage of FGL1 or PD-L1-positive IC among total IC (non-tumor cells) were quantified. We considered PD-L1 staining score of ≥ 5% on TC or IC as PD-L1 positivity, which was defined previously [26, 27]. For FGL1, the staining score of ≥ 10% on TC or IC was defined positive. CD8^+^ T cells and LAG-3^+^ cells were reported as the number of positive cells per mm^2^. For dichotomization of cases, the optimal cutoff values of CD8^+^ T cells and LAG-3^+^ cells on prognosis were defined as 10.7/mm^2^ and 4.9/mm^2^, respectively, which were determined using X-tile software (Yale University, New Haven, CT, USA) [28]. It is notable that we used density (cell/mm^2^) as counting unit to compare the expression of LAG-3, FGL1, PD-L1 and CD8 between tumor and adjacent normal tissue groups and investigated the associations among these markers.

### Statistical analysis

All statistical analyses were performed using SPSS software (23.0; IBM) and Graphs were created using the Prism software program (GraphPad 7 Software).The correlations between immune markers were performed using Spearman rho-rank function and the associations between immune marker expression and clinicopathologic variables were used the Chi-square or Fisher exact test. Differences of continuous variables between groups were determined using the Mann–Whitney U test and Wilcoxon Rank-Sum test. OS was calculated from the date of diagnosis to the date of death or the last known follow-up. Disease-free survival (DFS) was measured from the date of surgery to the date of recurrence, death, or the last follow-up. The survival curves were estimated using the Kaplan–Meier method and compared by the log-rank test. Multivariate analysis was performed by Cox regression to evaluate the independent prognostic factors. Two-sided *p* values < 0.05 were considered to indicate statistical significance.

## Results

### Expression of LAG-3, FGL1, PD-L1, and CD8^+^ T cells and their associations with clinicopathologic features in HCC

**Fig. 1:**
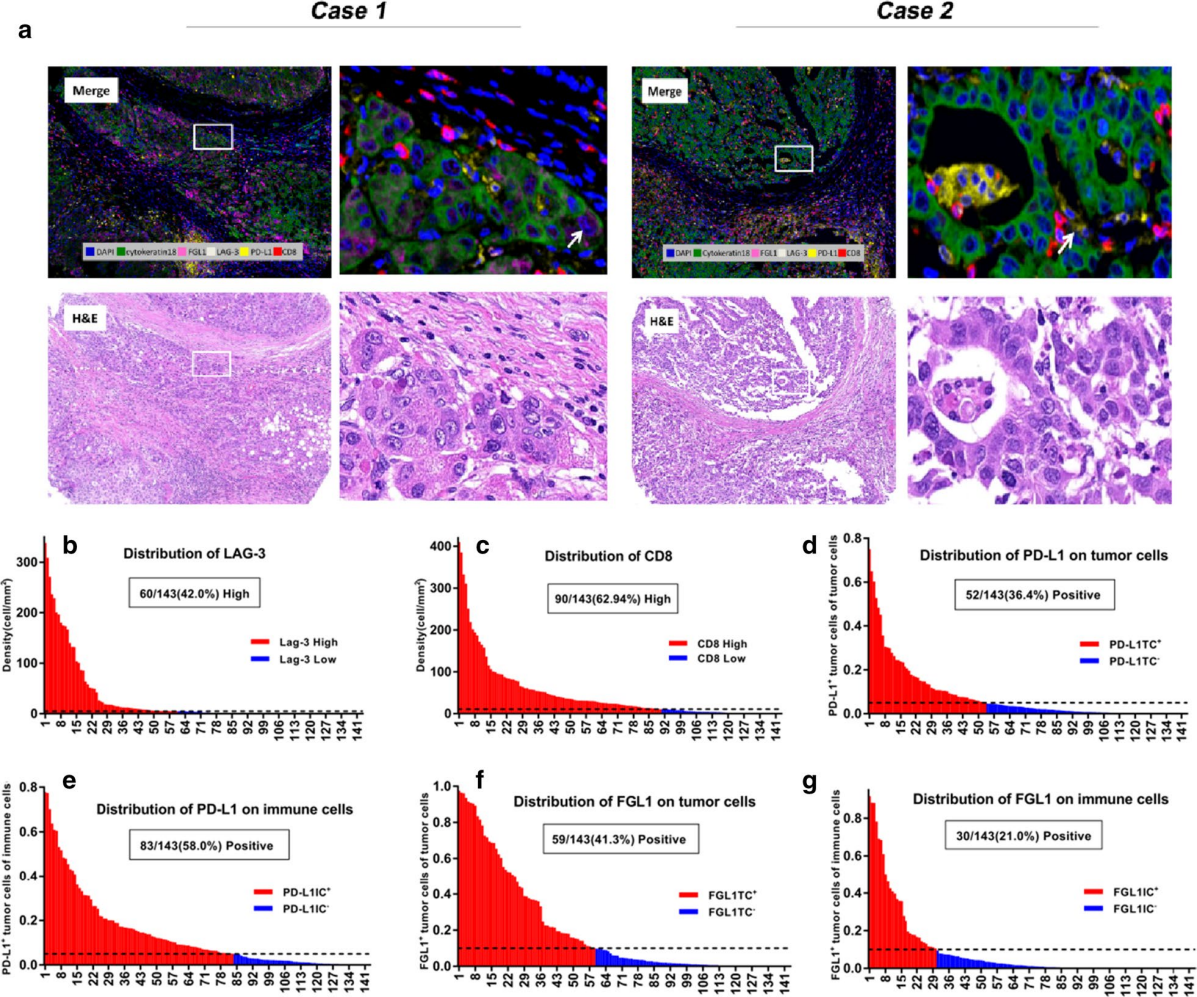
Opal six-colour multiplex immunofluorescence images of HCC tissues and levels of different immune targets in HCC. **a** Representative mIF and H&E staining images of HCC tissues. Arrow in case 1, Cytokeratin18^+^FGL1^+^ cells. Arrow in case 2, Cytokeratin18^+^PD-L1^+^ cells. DAPI was used to visualize nucleus (blue). Distribution of levels of LAG-3^+^ cells (**b**), CD8^+^ T cells (**c**), PD-L1 on TC (**d**), PD-L1 on IC (**e**), FGL1 on TC (**f**) and FGL1 on IC (**g**) in 143 HCC cases. The frequency of positive or high expression for each marker was displayed in parenthesis. The detailed cut-points used to define positive or high expression of these markers were described in methods. *N* negative, *P* positive, *TC* tumor cells, *IC* immune cells

Using mIF and image analysis approaches, we evaluated the location and abundance of LAG-3, FGL1, PD-L1, and CD8^+^ T cells in 143 pairs of HCC and matched non-tumor liver tissues. The representative 6-color mIHC staining images of HCC were showed in Fig. 1a. We applied H&E staining to validate the pathology type of HCC samples (Fig. 1a). The densities of LAG-3 and CD8 ranged from 0–338.36/ mm^2^ (median 2.53, mean 28.44) and from 0 to 409.76/mm^2^ (median 21.86, mean 46.29), respectively. 60 of the 143 cases (42.0%) were designated as high LAG-3^+^ cells (i.e.,LAG-3 number > 4.9/mm^2^) and 90 of the 143 cases (62.9%) as high CD8^+^ T cells (i.e.,CD8 number > 10.7/mm^2^) (Fig. 1b, c). PD-L1 was both recognized in TC and IC with cytoplasmic and membranous staining. 52 of 143 (36.4%) had positive PD-L1 TC staining (≥ 5%), and 83 of 143 (58.0%) had positive PD-L1 IC staining (≥ 5%) (Fig. 1d, e). We then evaluated the abundance of FGL1 expression on TC and IC. 59 of 143(41.3%) cases had positive FGL1 TC (≥ 10%) and 30 of 143 (21.0%) patients had positive FGL1 IC staining (≥ 10%) (Fig. 1f, g). The relationships between these immune markers’ expression and clinicopathological parameters were summarized in Table1. Positive PD-L1 expression on tumor cells (PD-L1 TC^+^) correlated positively with smaller tumor size (*P* = 0.008). Positive FGL1 expression on tumor cells (FGL1 TC^+^) correlated with a less Cirrhosis pattern (*P* = 0.002) and FGL1 IC^+^ was more frequent in patients with age older than 50 (*P* = 0.008).

### Correlation of LAG-3, FGL1, PD-L1 and CD8^+^ T cells expression

**Fig. 2:**
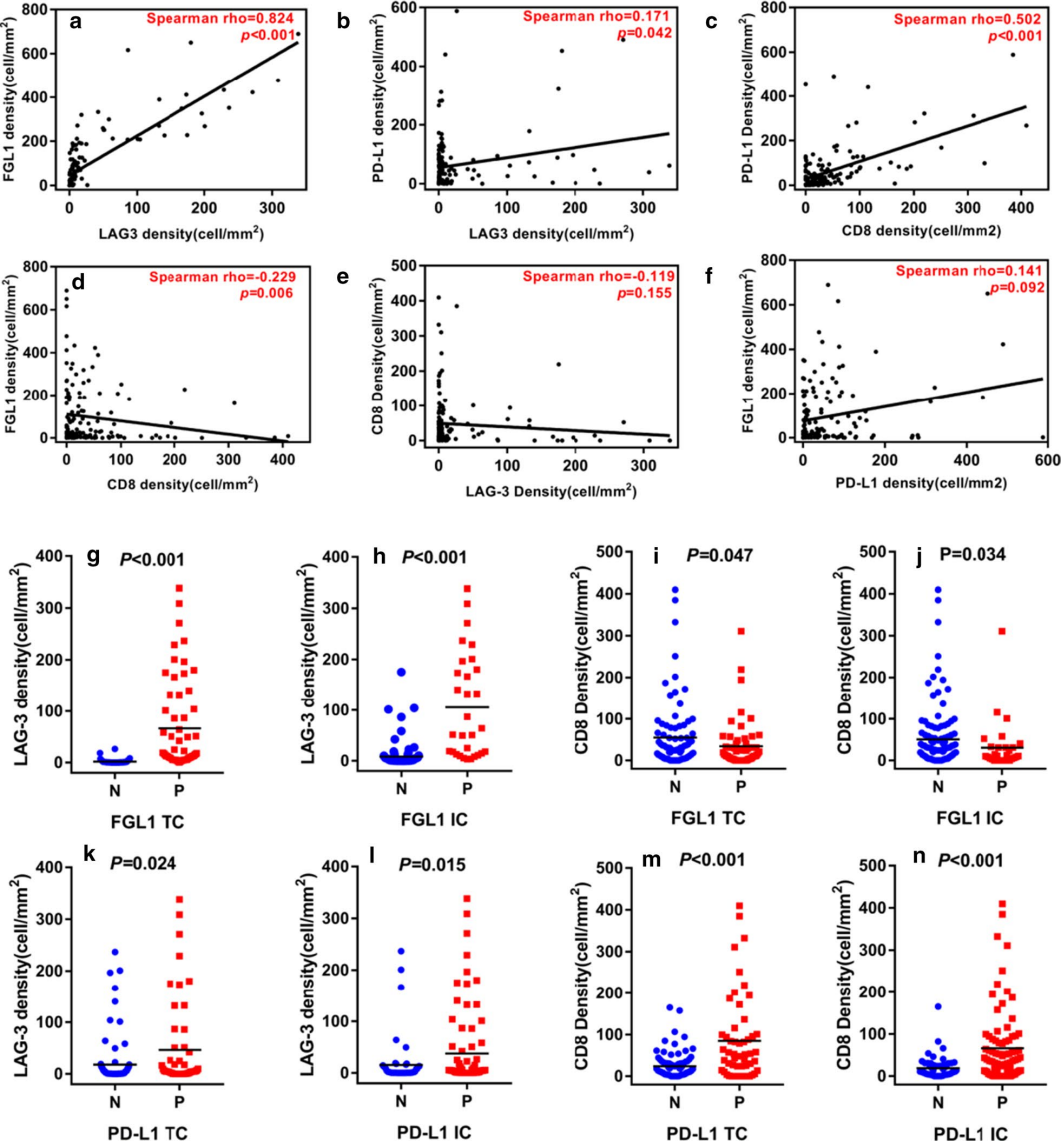
Correlation between the densities of LAG-3, FGL1, PD-L1 and CD8^+^ T cells and quantitative assessment of LAG-3^+^cells and CD8^+^ T cells according to FGL1 or PD-L1 status on TC and IC in HCC. Associations between the densities of LAG-3, FGL1, PD-L1 and CD8 in HCC by Spearman correlation analysis (**a**–**f**). The densities of LAG-3^+^cells were higher but that of CD8^+^ T cells were lower in FGL1 TC^+^ and FGL1 IC^+^ than in FGL1 TC^−^ and FGL1 IC^–^ respectively (**g**–**j**). Both the densities of LAG-3^+^cells (**k**, **l**) and CD8^+^ T cells (**m**, **n**) were greater in patients with PD-L1 TC^+^ and PD-L1 IC^+^ than in PD-L1 TC^−^ and PD-L1 IC^−^, respectively. *P* values were determined by the Wilcoxon Rank-Sum test. *TC* tumor cells, *IC* immune cells

**Fig. 3:**
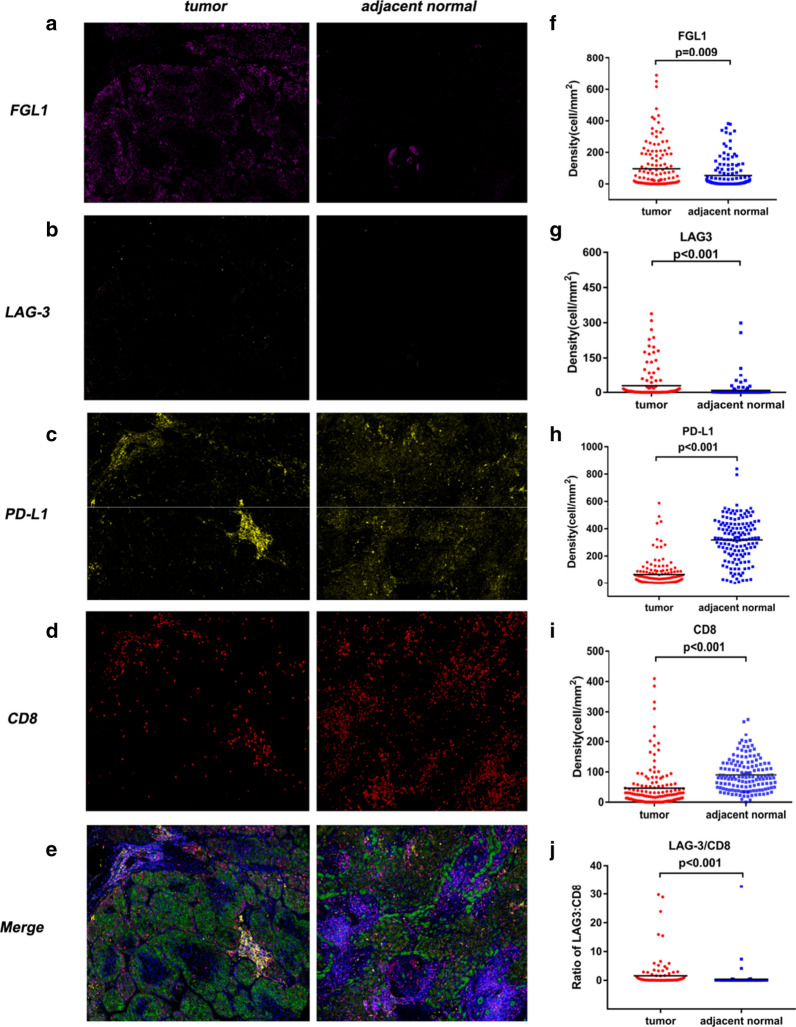
Comparison of densities of LAG-3, FGL1, PD-L1 and CD8^+^ T cells in HCC tissues and adjacent normal liver tissues. Representative images of FGL1, LAG-3, PD-L1, CD8 and six-colour merge in one pair of HCC case and adjacent normal liver tissue (**a**–**e**). The densities of FGL1 and LAG-3^+^cells were significantly increased in tumor tissues compared to adjacent normal tissues (**f**, **g**). The levels of PD-L1 and CD8^+^ T cells were significantly reduced in tumor samples compared to adjacent normal samples (**h**, **i**). The LAG-3/CD8 ratio was significantly elevated in tumor tissues compared to adjacent normal tissues (**j**). *P* values were determined by the Mann–Whitney *U* test

We investigated the associations between LAG3, FGL1, PD-L1 and CD8^+^ T cells expression. We found the densities of LAG-3^+^cells were positively correlated with levels of FGL1 (Spearman’s rho = 0.824, *P* < 0.001; Fig. 2a). LAG-3^+^cells densities also positively associated with PD-L1 expression but with much weaker correlation than the FGL1 (Spearman’s rho = 0.171, *P* = 0.042; Fig. 2b). Moreover, the numbers of CD8^+^ T cells were also positively correlated with the PD-L1 levels (Spearman’s rho = 0.502, *P* < 0.001; Fig. 2c) but negatively correlated with FGL1 expression (Spearman’s rho = − 0.229, *P* = 0.006; Fig. 2d). However, There was no significant correlation between the expression of CD8 and LAG-3, PD-L1 and FGL1 (Fig. 2e, f). We next compared the densities of LAG-3^+^cells and CD8^+^ T cells between the FGL1, PD-L1 status on TC and IC. We found the densities of LAG-3^+^cells were significant higher in positive FGL1 expression on tumor cells (FGL1 TC^+^) cases compared to negative FGL1 expression on tumor cells (FGL1 TC^−^) cases (*P* < 0.001), and in positive FGL1 expression on immune cells (FGL1 IC^+^) cases compared to negative FGL1 expression on immune cells (FGL1 IC^−^) cases (*P* < 0.001) (Fig. 2g, h). However, the CD8^+^ T cells densities were significant lower in FGL1 TC^+^ cases (*P* = 0.047) and FGL1 IC^+^ cases (*P* = 0.034) (Fig. 2i, j). Furthermore, the numbers of LAG-3^+^cells and CD8^+^ T cells were both greater in PD-L1 TC^+^ (LAG-3: *P* = 0.024; CD8: *P* < 0.001; Fig. 2k and m) and positive PD-L1 expression on immune cells (PD-L1 IC^+^) cases (LAG-3: *P* = 0.015; CD8: *P* < 0.001; Fig. 3l, n) than negative PD-L1 expression on tumor cells (PD-L1 TC^−^) and negative PD-L1 expression on immune cells (PD-L1 IC^−^) cases respectively.

### Differential expression patterns of LAG-3, FGL1, PD-L1, and CD8^+^ T cells and LAG-3/CD8 ratio in HCC tissues and paired adjacent normal tissues

Figure 3a−e showed representative stainings in tumor tissue and matched normal liver tissues. We found higher densities of LAG-3 and FGL1 in HCC tissues than in normal adjacent tissues (LAG3: 2.53 vs. 0.42 per mm^2^, *P* < 0.001; FGL1: 25.09 vs. 9.03 per mm^2^, *P* = 0.009; Fig. 3f, g). Conversely, there was a statistically significant underexpression of PD-L1 and CD8 in the tumor tissues than in corresponding normal liver tissues (CD8: 21.86 vs. 81.61 per mm^2^, *P* < 0.001; PD-L1:31.60 vs. 329.12 per mm^2^, *P* < 0.001; Fig. 3h, i).

Next, we evaluated the ratios of LAG-3 to CD8 densities. HCC tissues exhibited a significantly higher LAG-3/CD8 ratio compared with that in the adjacent normal liver tissues (Fig. 3j).These results indicated that the immunosuppressive molecules are expressed not only in the tumor but also in adjacent normal tissues of HCC patients with different expression patterns.

### Prognostic significance of LAG-3, FGL1, PD-L1, and CD8^+^ T cells expression

**Fig. 4:**
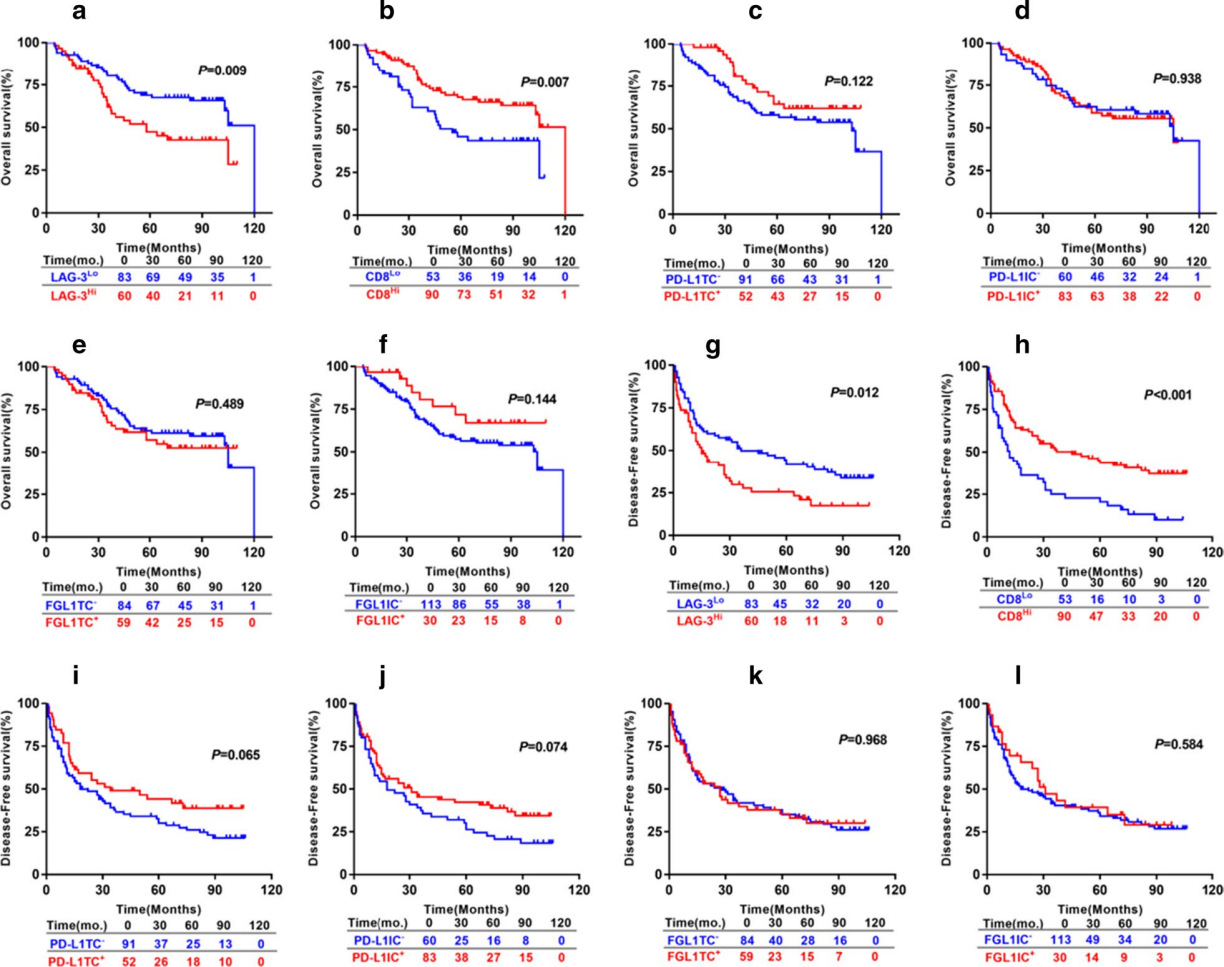
Prognostic significance of the levels of LAG-3^+^ cells, CD8^+^T cells, PD-L1 on TC, PD-L1 on IC,FGL1 on TC and FGL1 on IC in HCC patients. OS (**a**–**f**) and DFS (**g**–**l**) of HCC patients based on the levels of LAG-3^+^cells and CD8^+^T cells, PD-L1 and FGL1 status on TC and IC. Survival curves were assessed by the Kaplan–Meier method, and differences between groups were evaluated using log-rank test. The number of patients at risk was reported. *TC* tumor cells, *IC* immune cells

**Fig. 5:**
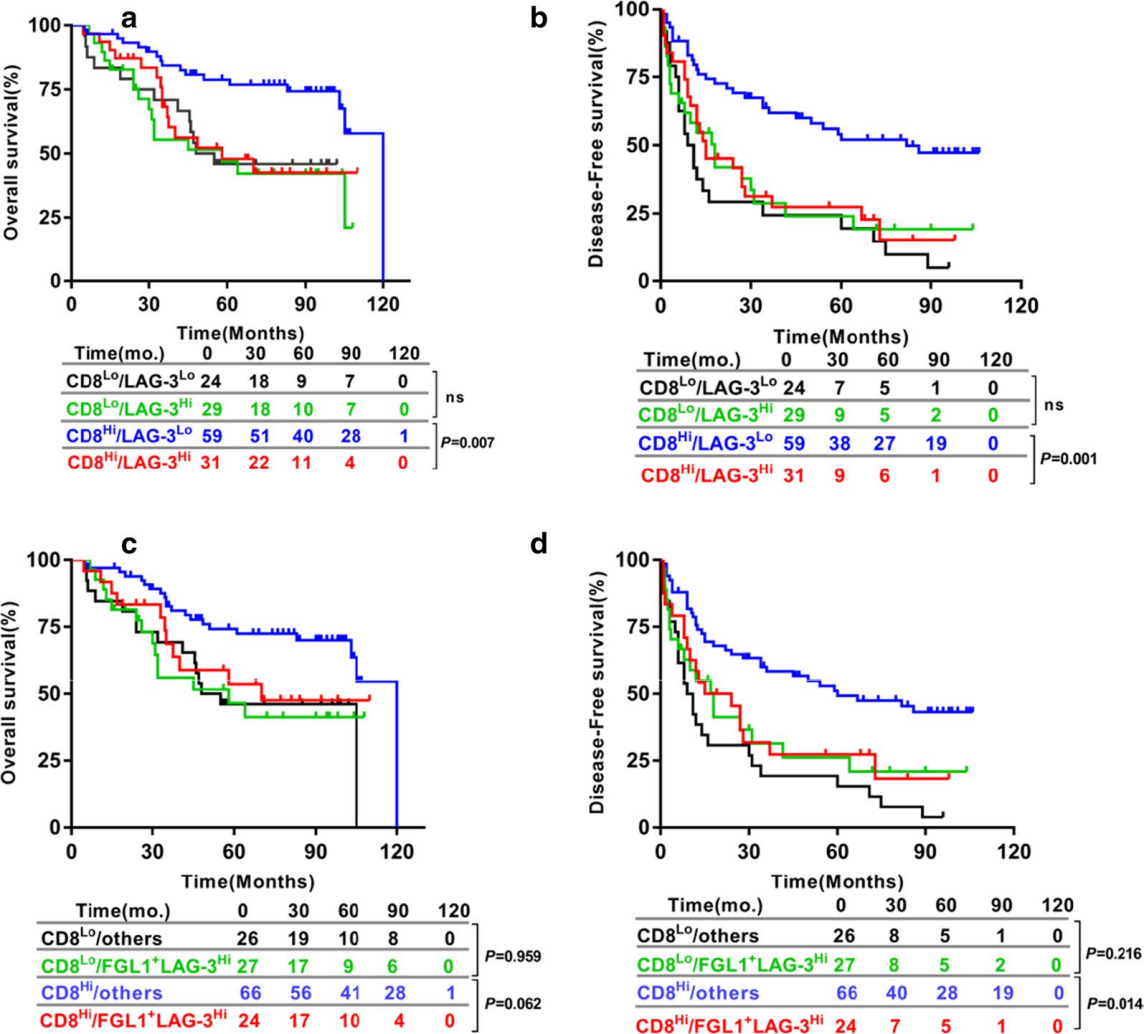
HCC-specific OS and DFS of LAG-3^+^cells levels or plus FGL1 status on TC in relation to CD8^+^T cells densities in the combined cohorts. Kaplan–Meier analysis of OS (**a**) and DFS (**b**) based on the combination of the levels of LAG-3^+^cells and CD8^+^T cells. HCC-specific OS (**c**) and DFS (**d**) of LAG-3^+^cells levels plus FGL1 status on TC in relation to CD8^+^T cells densities in the combined cohorts using Kaplan–Meier analysis. Others: the patients who are either high levels of LAG-3^+^cells or positive FGL1 status on TC or both low densities of LAG-3^+^cells and negative FGL1 status on TC. Differences in survival were analyzed by log-rank test. The number of patients at risk was reported. *TC* tumor cells, *IC* immune cells, *OS* Overall survival, *DFS* Disease-free survival

For 143 HCC patients, the 5 year OS rate was 59.6% and 5 year DFS rate was 35.2%. The patients with large numbers of LAG-3^+^cells had a significantly shorter OS (*P* = 0.009; Fig. 4a) and DFS (*P* = 0.012; Fig. 4g). The patients having a high densities of CD8^+^ T cells were correlated with a significantly improved OS (*P* = 0.007; Fig. 4b) and DFS (*P* < 0.001; Fig. 4h). PD-L1 TC^+^ were associated with a tendency towards improved OS (*P* = 0.122; Fig. 4c) and DFS (*P* = 0.065; Fig. 4i) than PD-L1 TC^−^. PD-L1 IC^+^ had a tendency towards improved DFS (*P* = 0.074; Fig. 4j), but not associated with OS (*P* = 0.938; Fig. 4d). DFS and OS did not statistically correlate with FGL1 expression on either TC and IC (Fig. 4e, f, k and l). We continued to assess the prognosis of FGL1-LAG-3 pathway based on the CD8 expression. We found patients with high numbers of LAG-3^+^cells (LAG-3^Hi^) had shorter OS (*P* = 0.007; Fig. 5a) and DFS (*P* = 0.001; Fig. 5b) than patients with low numbers of LAG-3^+^cells ( LAG-3^Lo^) in high CD8^+^ T cell expression(CD8^Hi^) group but did not hold true in the low CD8^+^ T cell expression (CD8^Lo^) patients. Furthermore, LAG-3^Hi^/FGL1TC^+^ patients showed an inferior DFS (*P* = 0.014; Fig. 5d) and a tendency towards shorter OS (*P* = 0.062; Fig. 5c) than patients who were either LAG-3^Hi^ or FGL1 TC^+^ or both LAG-3^Lo^ and FGL1 TC^−^ in CD8^Hi^ group but not CD8^Lo^ subset. Next, the parameters with *P* values < 0.1 from univariate analysis were entered into the multivariate cox proportional hazards analysis. Multivariate analysis revealed that expression of LAG-3^+^cells and CD8^+^ T cell, cirrhosis, vascular invasion and tumor size were independent prognostic factors for OS. We also found that the levels of LAG-3^+^cells and CD8^+^ T cells, HbsAg, cirrhosis, tumor size and tumor number were independent predictors of DFS (Table 2).

**Table 2:** Univariate and multivariate analysis for OS

Variables	OS			PFS		
	Univariate	Multivariate	*p* value	Univariate	Multivariate	*p* value
	*p* value	HR(95% CI)		*p* value	HR(95% CI)	
Age, years(> 50 vs. ≤ 50)	0.911		NA	0.713		NA
Gender(male vs. female)	0.873		NA	0.115		NA
HBsAg (positive vs. negative)	0.065		NS	0.001	3.717(1.504–9.185)	0.004
AFP, ng/ml (> 20 vs. ≤ 20)	0.094		NS	0.262		NA
Cirrhosis (yes vs. no)	0.036	2.098(1.240–3.550)	0.006	0.018	2.217(1.441–3.412)	0.000
Tumor number (multiple vs. single)	0.122		NA	0.001	1.530(1.182–1.981)	0.001
Tumor size, cm (> 5 vs. ≤ 5)	0.005	1.893(1.059–3.384)	0.031	0.000	2.690(1.693–4.275)	0.000
Tumor differentiation (III vs. I-II)	0.124		NA	0.111		NA
vascular invasion (present vs. absent)	0.000	4.171(1.830–9.504)	0.001	0.038		NS
LAG-3 expression (high vs. low)	0.009	1.879(1.080–3.267)	0.025	0.012	1.642(1.058–2.549)	0.027
CD8 expression (high vs. low)	0.007	0.562(0.330–0.957)	0.034	0.000	0.618(0.400–0.956)	0.031
PD-L1 status on TC (positive vs. negative)	0.122		NA	0.065		NS
PD-L1 status on IC (positive vs. negative)	0.938		NA	0.074		NS
FGL1 status on TC (positive vs. negative)	0.489		NA	0.968		NA
FGL1 status on IC (positive vs. negative)	0.144		NA	0.584		NA

*P* < 0.05 was considered as statistically significant

*HOS* overall survival, *DFS* disease-free survival, *HBsAg* hepatitis B surface antigen, *AFP* αfetoprotein, *HR* hazard ratio, *CI* confidential interval, *NA* not adopted, *NS* not significant, *TC* tumor cells, *IC* immune cells

Because LAG-3 had synergistic effects with PD-1/PD-L1,we wondered whether combining LAG-3 with PD-L1 would predict the prognosis. Additional file 1: Figure S2 showed that PD-L1 TC^+^ cases with low numbers of LAG-3^+^cells were associated with best OS and DFS, while PD-L1 TC^−^ cases with high numbers of LAG-3^+^cells were associated with the worst OS and DFS. The patients with LAG-3^Lo^/PD-L1 TC^+^ or LAG-3^Hi^/PD-L1 TC^−^ were associated with intermediate OS and DFS.

## Discussion

Using multiplex immunofluorescence, we determined the levels, associations, and clinical significances of LAG-3, FGL1, PD-L1 and CD8^+^ T cells in human HCC. Previous studies have demonstrated that LAG-3 is upregulated in various types of tumor and suppresses the proliferation, activation and effector functions of T cells [19]. Due to the immune suppressive function similar to PD-1, the LAG-3 is assessed in many clinical trials for their antitumor ability. However, in contrast to PD-L1, the major ligand for LAG-3 which mediates its immune inhibitory functions remains controversial. Galectin-3 and LSECtin have been shown to interact with LAG-3 and negatively regulate T cell function, but they have several other binding proteins [29–32] and induce T cell suppression without the engagement of LAG-3 [33]. MHC class II, identified as the first and most recognized ligand for LAG-3. However, some antibody can promote T cell functions in several tumor models, despite the fact that it blocks the LAG-3 D2 domain instead of the LAG-3 D1 domain which directly interacts with MHC class II [34–36]. Recent research shows that FGL1 is an alternative high-affinity ligand for LAG-3 and anti-FGL1 mAb lacks its original antitumor effects when deficiency of LAG-3 [18]. Despite previous encouraging results, data on FGL1-LAG-3 pathway in HCC are lacking.

Here, we found high expression of LAG-3^+^cells was seen in 42% of HCC, the variable results were reported ranging 11–65% when using different cut-points [37–40]. In addition, a highly significant concordance of the densities of LAG-3^+^cells with the expression of FGL1 confirmed the high-affinity between LAG-3 and its ligand—FGL1. Moreover, we found high levels of LAG-3^+^cells were independent predictor of poor OS and DFS. Similar to our result, high expression of LAG-3 predicted poor survival in head and neck squamous cell carcinoma [41], melanoma [37], soft tissue sarcomas [42] and non-small cell lung cancer (NSCLC) [40]. On the contrary, other studies in esophageal squamous cell carcinoma [43], breast cancers [39] and NSCLC [44] show the opposite results, and only one study in HCC has showed no prognostic significance [45]. Differences in patient cohorts, disease types, and selection of cutoffs can contribute to the discrepancy. It is widely established that elevated levels of cytotoxic CD8^+^ T cells are associated with stronger anti-tumor effect and improved prognosis in human cancers, and LAG-3 has served as inhibitory molecule to attenuate the effector function of CD8^+^ T cells in HCC [46]. Consistent with previous studies, we confirmed that CD8^Hi^ was correlated with a good OS and DFS, furthermore, LAG-3^Hi^ patients had inferior OS and DFS than LAG-3^Lo^ patients in CD8^Hi^ subset but did not hold true in the CD8^Lo^ group. The similar tendency was detected when combination of LAG-3 and FGL1: LAG-3^Hi^/FGL1 TCs^+^ patients had a shorter DFS and a tendency towards inferior OS than patients who were either LAG-3 high or FGL1 TCs^+^ or both LAG-3 low and FGL1 TCs^−^ in CD8^Hi^group but not CD8^Lo^ patients. Taken together, our findings may support that the immune-inhibitory functions of LAG-3 or FGL1-LAG-3 pathway were dependent on CD8^+^ T cells. The recent study has also demonstrated that the anti-tumor effects of anti-FGL1 or anti-LAG-3 mAb are depending on CD8^+^ T cells [18].

PD-L1 TC^+^ were detected in 36.4% of HCC when using cut-off value of 5% in our research. Two previous studies report PD-L1TC^+^ in 29.8% and 19% of HCC cases using the same cut-point, respectively [47, 48]. Here, our finding did not observe significant correlation between the expression of PD-L1 on TC or IC and survival in multivariate analysis, but a tendency towards improved OS in PD-L1 TC^+^ patients. The opposite findings are reported in several prior studies in HCC [49, 50], while results from other studies are consistent with our results [48, 51]. These contradictions can be as a result of variations in antibodies, detection technique and cases analyzed. Therefore, it is critical to develop a uniform standard regarding PDL1 expression to interpret its significance in TME more accurately.

Further, we analyzed the cross-relationships between LAG-3, FGL1, PD-L1 and CD8^+^ T cells. There was no significant association between the densities of LAG-3 and CD8, FGL1 and PD-L1. CD8^+^ T cells densities were positively associated with PD-L1 TC^+^ and PD-L1 IC^+^ but negatively associated with FGL1 TC^+^ and FGL1 IC^+^. Moreover, the levels of PD-L1 showed much stronger positive correlation with the numbers of CD8^+^ T cells than LAG-3^+^ cells in current study, and previous research has suggested that PD-L1 can be induced by IFN-γ secreted by CD8^+^T cells in HCC [48]. Taken together, these findings demonstrated that FGL1-LAG-3 axis and PD-L1 might be biomarkers of active and suppressed immune microenvironment, respectively, which were consistent with their opposite prognosis.

Recent studies demonstrate that LAG-3 may have synergistic action with PD-1/PD-L1 and the combination of anti-LAG-3 mAb and anti-PD-1 mAb exhibits exciting effects in tumors which are resistant to previous PD-L1 blocker [52]. So we wondered whether combining LAG-3 with PD-L1 would predict the prognosis. Our results demonstrated that LAG-3^Hi^/PD-L1 TC^−^ patients had a shorter DFS and OS than LAG-3^Lo^/PD-L1 TC^+^ patients. Increasing evidences have suggested that patients with positive expression of PD-L1 are more likely to benefit from anti-PD-1/PD-L1 therapy, and the patients with high LAG-3 expression (≥ 1%) tend to respond to the immunotherapy [52]. Therefore, we inferred the LAG-3^Hi^/PD-L1TC^−^ patients with worst prognosis might benefit from the anti-LAG-3 mAb or the combination of anti-LAG-3 and anti-PD-L1 mAb.

There are limitations to this study. First, it is a retrospective and single-center design. Although long-term follow-up enable our results more powerful, additional prospective studies with large cohort of patients are needed to confirm our conclusion. Second, the TMAs are just a sampling of the original tumor tissue, which can not reflect the completed information of these immune markers analyzed in the TME.

## Conclusions

In summary, our study indicated the variable distribution and functions of LAG-3, FGL1, PD-L1 and CD8^+^T cells in the TME, and these immune markers displayed distinct correlation with each other. Expression of LAG-3 and CD8 represented unfavorable and favorable prognostic biomarkers for HCC, respectively.

## Supplementary information

**Additional file 1: Figure S1.** H&E staining of the two TMAs. **Figure S2.** HCC-specific OS and DFS of LAG-3^+^cells levels in relation to PD-L1 status on TC in the combined cohorts.

**Additional file 2: Table S1.** Detailed information of primary antibody.

## Data Availability

The data generated or analyzed during the current study are included in this published article and its additional files.

## Abbreviations

mIF: Multiplex immunofluorescence
PD-L1: Programmed death-ligand1
CD8^+^Tcells: Cytotoxic T cells
LAG-3: Lymphocyte activating gene 3
FGL1: Ffibrinogen-like protein 1
TC: Tumor cells
IC: Immune cells
HCC: Hepatocellular carcinoma
OS: Overall survival
DFS: Disease-free survival
