# Supplementary material for: Expression and clinical significance of LAG-3, FGL1, PD-L1 and CD8+T cells in hepatocellular carcinoma using multiplex quantitative analysis
Source: J Transl Med. 2020 Aug 6;18:306. doi: 10.1186/s12967-020-02469-8 (PMC7409704; doi:10.1186/s12967-020-02469-8)
Supplement: Supplementary file 1 — Additional file 1: Figure S1. H&E staining of the two TMAs. Figure S2. HCC-specific OS and DFS of LAG-3+cells levels in relation to PD-L1 status on TC in the combined cohorts. [file 12967_2020_2469_MOESM1_ESM.docx]

**
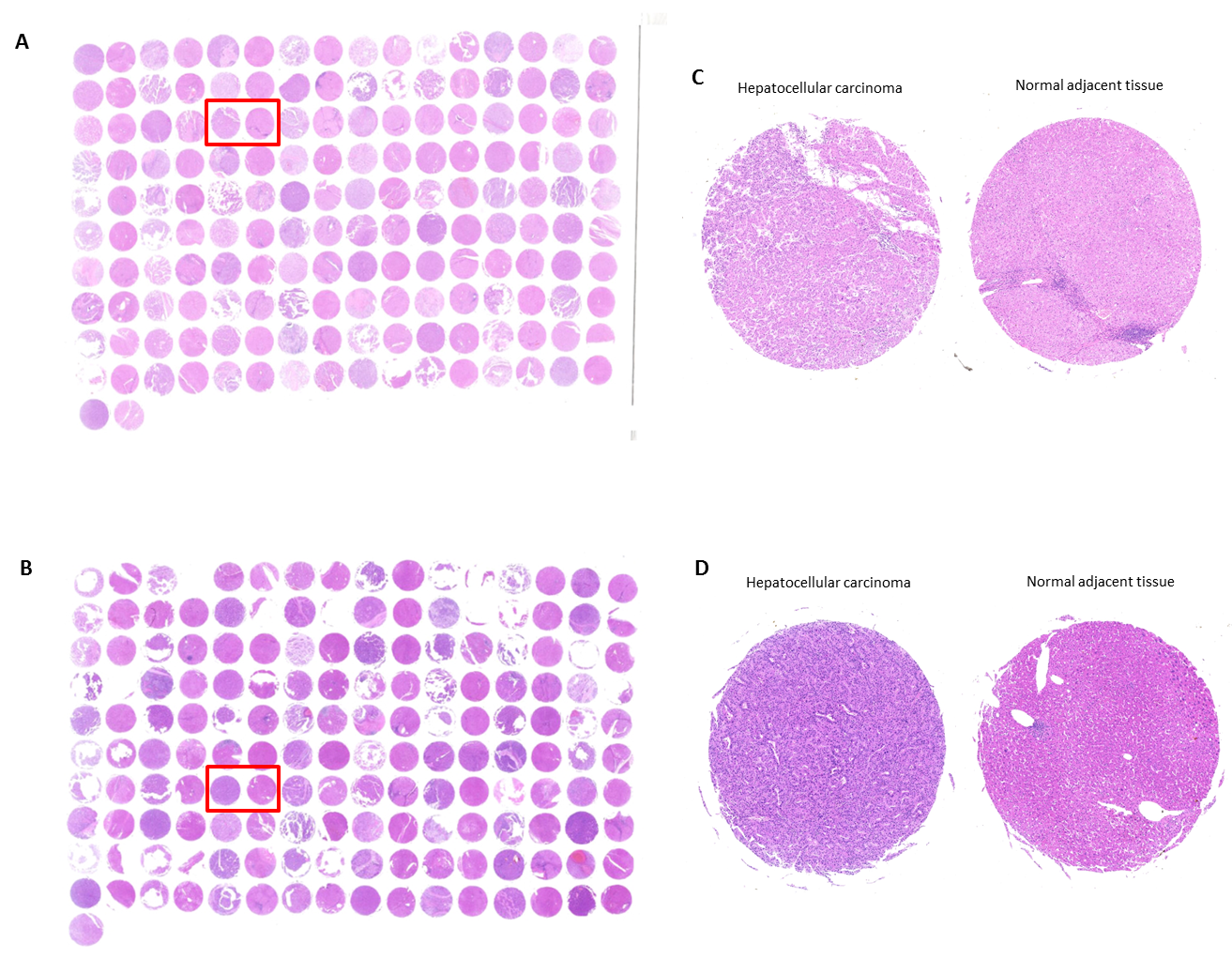
**

**Supplementary Figure S1: H&E staining of the two TMAs.**

**
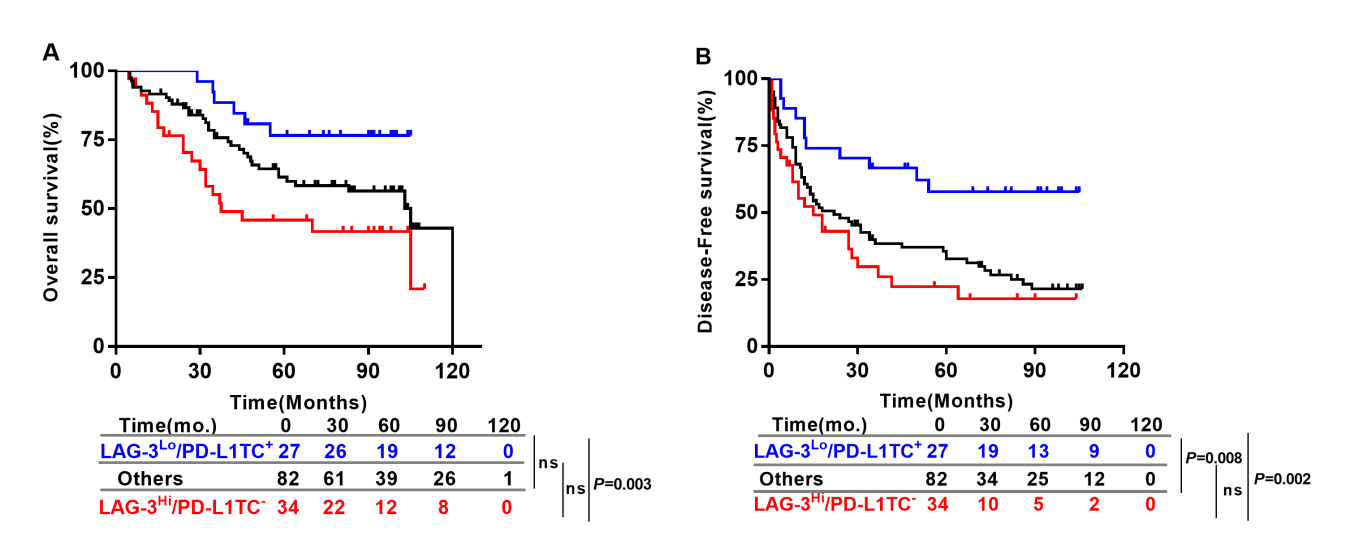
**

**Supplementary Figure S2: HCC-specific OS and DFS of LAG-3^+^cells levels plus**

### PD-L1 status on TC in the combined cohorts.

### Combination of the densities of LAG-3^+^cells and PD-L1 status on TC in relation to HCC-specific OS (A) and DFS (B) using Kaplan-Meier analysis. Differences in survival were analyzed by log-rank test. Others: the patients with low densities of LAG-3^+^cells and negative PD-L1 status on TC or high densities of LAG-3^+^cells and positive PD-L1 status on TC. The number of patients at risk was reported. Abbreviations: TC, tumor cells; IC, immune cells.
