# Supplementary material for: Expression and clinical significance of LAG-3, FGL1, PD-L1 and CD8+T cells in hepatocellular carcinoma using multiplex quantitative analysis
Source: J Transl Med. 2020 Aug 6;18:306. doi: 10.1186/s12967-020-02469-8 (PMC7409704; doi:10.1186/s12967-020-02469-8)
Supplement: Supplementary file 2 — Additional file 2: Table S1. Detailed information of primary antibody. [file 12967_2020_2469_MOESM2_ESM.docx]

**Supplementary Table 1: Detailed information of primary antibody**

| Position | Fluorophore | Antibody | Dilution | Catalog number | Brand name |
| --- | --- | --- | --- | --- | --- |
| 1 | Opal 520 | Cytokeratin 18 | 1:1000 | A01357-1 | Boster |
| 2 | Opal 540 | LAG-3 | 1:500 | Ab209236 | Abcam |
| 3 | Opal 570 | FGL1 | 1:500 | Ab197357 | Abcam |
| 4 | Opal 650 | PDL1 | 1:500 | 13684s | [Cell Signaling Technology](http://www.baidu.com/link?url=gPjWj_IvsweMG85lYd1mvB74lMAobXEHmJJAarN3ZdvZMYdOx5UFbcWS9IehyxdL) |
| 5 | Opal 690 | CD8 | 1:500 | 70306s | [Cell Signaling Technology](http://www.baidu.com/link?url=gPjWj_IvsweMG85lYd1mvB74lMAobXEHmJJAarN3ZdvZMYdOx5UFbcWS9IehyxdL) |

Abbreviations:FGL1, Fibrinogen-like protein 1; LAG-3, Lymphocyte activating gene 3; PD-L1, programmed cell death-ligand 1; CD8, cluster of differentiation 8.
